# Supplementary figures and images for: Nutritional status positively impacts humoral immunity against its Mycobacterium tuberculosis, disease progression, and vaccine development
Source: PLoS One. 2020 Aug 6;15(8):e0237062. doi: 10.1371/journal.pone.0237062 (PMC7410285; doi:10.1371/journal.pone.0237062)

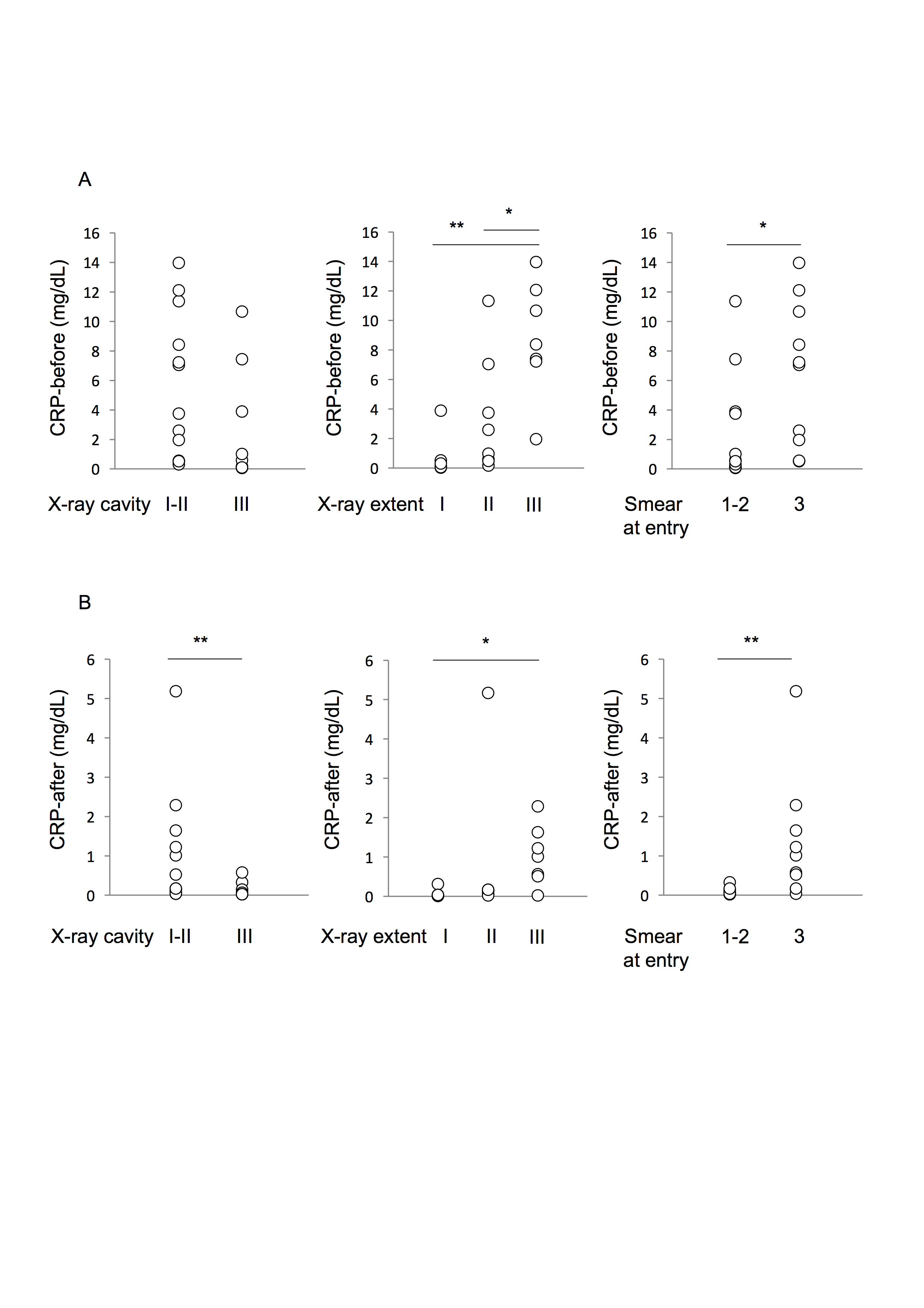

Supplement: S1 Fig — (A) Correlation between serum CRP before treatment and disease severity at onset. (B) Correlation between serum CRP after treatment and disease severity at onset. For statistical analyses, groups I and II of “X-ray cavity” and groups 1 and 2 of “Smear at entry are combined because of the small sample number of patients diagnosed as group I in “X-ray cavity” and 1 in “Smear at entry”. **: p < 0.01, *: p < 0.05. (TIFF) [file pone.0237062.s001.tiff]

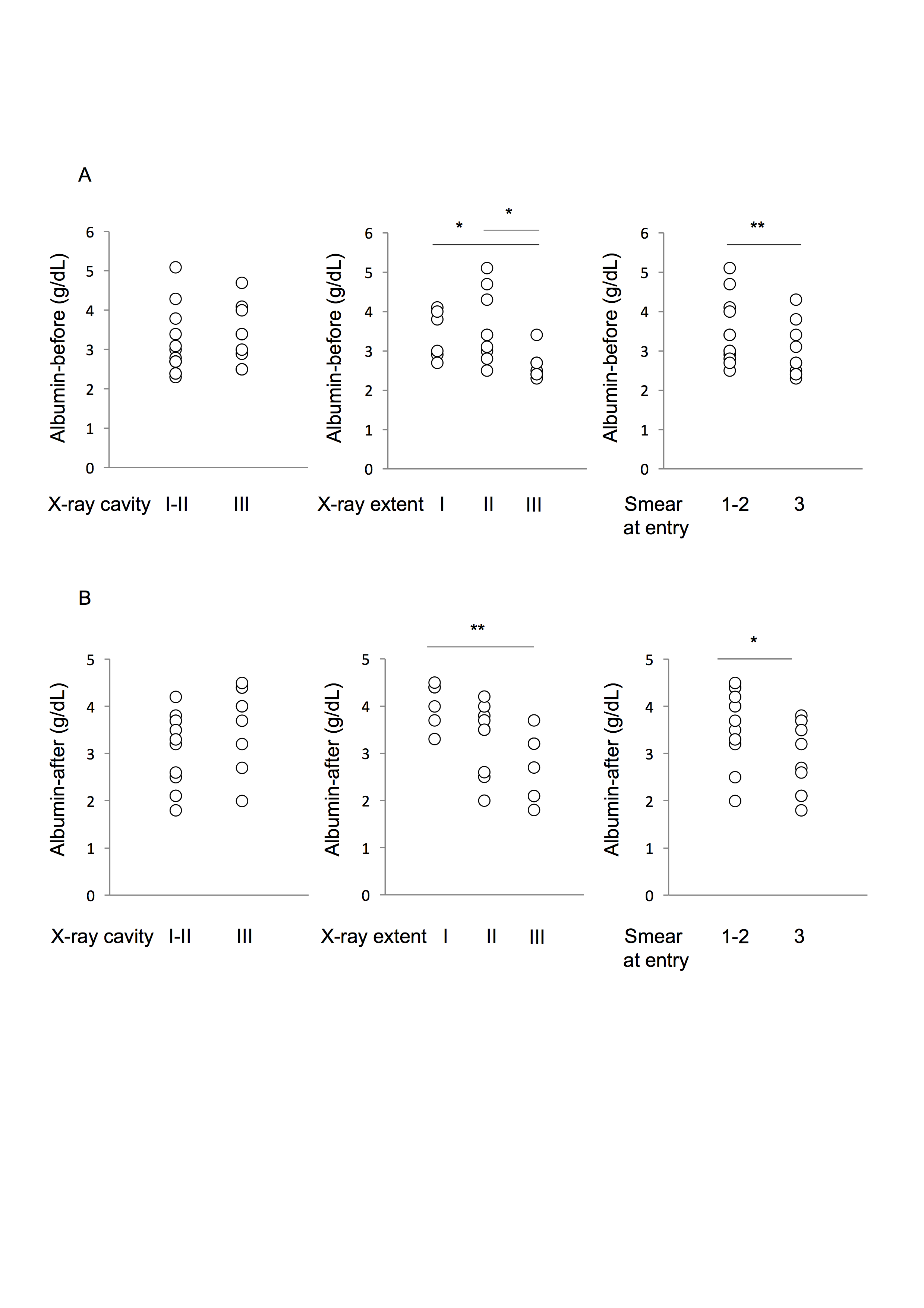

Supplement: S2 Fig — (A) Correlation between serum albumin before treatment and disease severity at onset. (B) Correlation between serum albumin after treatment and disease severity at onset. **: p < 0.01, *: p < 0.05. (TIFF) [file pone.0237062.s002.tiff]
