# Supplementary material for: Nutritional status positively impacts humoral immunity against its Mycobacterium tuberculosis, disease progression, and vaccine development
Source: PLoS One. 2020 Aug 6;15(8):e0237062. doi: 10.1371/journal.pone.0237062 (PMC7410285; doi:10.1371/journal.pone.0237062)
Supplement: Attachment — Submitted filename: PLoS+ONE+response.docx [file pone.0237062.s003.docx]

30^th^ April, 2020

Professor Fredrick Quinn,

Academic Editor of PLOS ONE

Re: PONE-D-19-26473

Dear Prof. Fredrick Quinn,

We appreciate the opportunity to revise our original manuscript entitled “**Nutritional status positively impacts humoral immunity against its *Mycobacterium tuberculosis*, disease progression, and vaccine development.**”. The original manuscript has been dramatically modified according to the reviewers’ comments and suggestions, and we believe the revised manuscript will be more informative for readers of your journal.

Our responses to the reviewers’ comments are addressed in detail below.

Changes in the revised manuscript are in red.

Again, we are grateful for the opportunity to submit a manuscript for publication in PLOS ONE.

Sincerely Yours,

Yoshihiko Hoshino, MD, PhD

Department of Mycobacteriology National Institute of Infectious Diseases, Higashi-Murayama, Tokyo, JAPAN

We wish to thank the reviewer for the insightful comments and suggestions on our paper. The comments have helped us improve the paper.

1. Please provide additional details regarding participant consent. In the ethics statement in the Methods and online submission information, please ensure that you have specified (1) whether consent was informed and (2) what type you obtained (for instance, written or verbal, and if verbal, how it was documented and witnessed). If your study included minors, state whether you obtained consent from parents or guardians. If the need for consent was waived by the ethics committee, please include this information.

Informed consent was obtained as a written form. We added the information in line 101, page 6.

2. We note that you have included the phrase “data not shown” in your manuscript. Unfortunately, this does not meet our data sharing requirements. PLOS does not permit references to inaccessible data. We require that authors provide all relevant data within the paper, Supporting Information files, or in an acceptable, public repository. Please add a citation to support this phrase or upload the data that corresponds with these findings to a stable repository (such as Figshare or Dryad) and provide and URLs, DOIs, or accession numbers that may be used to access these data. Or, if the data are not a core part of the research being presented in your study, we ask that you remove the phrase that refers to these data.

In accordance to the reviewer #1’s suggestion, we have removed the data obtained from LTBI patients and the phrase “data not shown” that refers to the data corresponding to the serum nutritional levels of LTBI patients.

Answers to the comments

Reviewer #1

We strongly appreciate the reviewer’s comments and suggestions on this paper.

Answers to the specific comments:

1. Please provide a reason not to include nutritional data from patients used in your earlier published studies. This would increase the number of patients and benefit the statistical analysis.

As the blood sample volumes obtained from the patients were low and limited, we could not afford to determine the serum biochemical parameters in addition to the analysis of serum antibody levels. In this study, we collected only the samples with enough volume for the measurement of serum nutritional status and the antibody levels at the same time.

2. Please provide more demographic details about the 22 patients enrolled in this study.

In accordance to the reviewer’s comment, we have added the demographic details about the patients to the line 104, page 6.

3. Please consider either adding more LTBI patients or removing those patients from the study. Only 6 data points is not enough to come to any conclusion.

According to the reviewer’s comment, we have removed LTBI patients from this study.

4. IS there any correlation between serum albumin or HDL versus TNFa levels in active TB patients?

We agree to make an analysis between HDL and inflammatory cytokines such as TNFa. Unfortunately, we have not determined the serum TNF-alpha levels in this study. As stated in 1, we only collected small volume of samples.

5. Please provide Fig 5B.

We are sorry not including Fig 5B now we have attached Fig 5B in the file.

Reviewer #2

Firstly and most importantly, the topic is too big for a small scale of study. As stated, 22 patients and 6 patients from 2 hospital, may not be sufficiently supportive for the correlation study. I also doubt about the statistical analysis for such small number of patients. I strongly recommend the authors to perform the study on a larger scale, on the number of both medical centers and patients.

We agree to the reviewer #2. If we could expand the numbers however, we are afraid we could not, which is a limitation of this study.

Secondly, a lot of the results and conclusions are based on only observational and descriptive data. For example, it’s pretty easy to perform such study, by describing what data and thus correlation from such results. However, how to further confirm and verify, still remains unclear in this study. For example, could the authors provide evidence on the correlation as concluded, by changing the lipid level then obtaining the change in TB development?

We really agree to the reviewer. However, this is just observational and descriptive study. Further study and analysis should be required, which we completely agree.

Thirdly, one slight drawback is, since the authors had stated the nutritional status, however, as seen from the section of Materials and Methods, the authors only stated all patients will follow the standard treatment in Japan for TB. Then how about the diets, which will eventually change their nutritional status? Consequently, detailed description are required to support the results and conclusions.

As far as diets, we did not evaluate these items. All patients were given regular meals. No special diets were considered.
