# Supplementary material for: Nutritional status positively impacts humoral immunity against its Mycobacterium tuberculosis, disease progression, and vaccine development
Source: PLoS One. 2020 Aug 6;15(8):e0237062. doi: 10.1371/journal.pone.0237062 (PMC7410285; doi:10.1371/journal.pone.0237062)
Supplement: Attachment — Submitted filename: Point by Point_200630_rev3.docx [file pone.0237062.s004.docx]

We wish to thank the reviewer for the insightful comments and suggestions on our paper. The comments have helped us improve the paper.

Answers to the specific comments.

*1. Line 52 "obesity is protective against" please provide a reference.*

→In accordance to the reviewer’s comment, we have added the references to the text.

*2. Line 104 demographics – Since gender cannot be in fractions please provide the exact number of males and females in the study.*

→We have provided the exact number of males and females to the text.

*3. Line 113 – 115 – X ray results – Is it possible to keep the scoring system consistent for X ray type and extent? Keep it either in ascending or descending order? Similarly change the figure S1? It is otherwise very confusing.*

→We completely agree with a reviewer however, this scoring system was established in 1959 and was long time used as a standard scoring system in Japan. Again, we agree that the system is a little bit curious and confusing but we cannot modify the system by ourselves without any permission.

*4. Line 118 and 119 – It is not clear what is +2 and +3? Is it >10 AFB per 100x field for +2? For +3, please explain more than 10 AFB per field. What is per field?*

→This is according to “diagnostic standards and classification of tuberculosis and other mycobacterial diseases (American Review of Respiratory Disease 1981, 123(3), 343–358). The scale was determined by Ziehl-Neelson staining, and per field means x1,000 field by an optical microscope (in the literature described above, there was an explanation as “Examination at 800-1000X is assumed”).

In conversion of the conventional Gaffky scale, ± accounts for G1 (conventional Gaffky scale), 1+; G2, 2+; G5, 3+; G9.

*5. Line 153 and 173 please provide the details of the statistical analysis applied to "Before" and "After" samples since they are paired.*

→We have added the description about statistical analysis in the text.

*6. Figure 1 and 2 - please position the "Before" label correctly on the graph.*

→As requested, we have corrected the position of the “Before” labels in the graphs.

*7. Figure 2 - What is the rationale behindshowing the IgG ELISA data as absorbance 450 nm and IgA ELISA data as ELISA index? Please consider a uniform data presentation.*

→We thank the reviewer’s comment on this point. Since we had to measure the antibody levels to multiple Mtb antigens using samples with limited volume, we evaluate IgG levels at the serum concentration as little as possible to save the samples. However, serum IgA levels are much lower than IgG when measured by the same method for the identification of serum IgG levels, we introduced the ELISA Index to evaluate the IgA levels in our previous study and the present manuscript according to the previous reports describing similar experiments. We have added the references to the text.

*8. Line 177 and 178 - please remove the p values since they are not indicated in the actual graph (fig. 2).*

→We have removed the p values from the legend.

*9. For Figure 1, 2 and 3 legends - Instead of writing what was done, it would help to write the results / observations.*

→As suggested, we have added the brief descriptions about the results to the legends.

*10. Line 177 and 188 - for statistics please write either p or P. Keep it uniform.*

→In accordance to the reviewer’s comment, we standardize the description of p-values in the text. Accordingly, we have also changed the “P” value to “p” in Fig. 3.

*11. Please provide figure 4C and 4D.*

→We appreciated this comment and corrected as the attachment.

*12. Line 202 - change TB patents to TB patients.*

→We have corrected the typo.

*13. Line 216 (Fig S1)- There is a difference between serum CRP levels before and after treatment. Just add a line stating that this is as expected. With decrease in disease severity with treatment, the serum CRP levels will decrease too.*

→In accordance to the reviewer’s suggestion, we have changed the text as follows: As expected, serum CRP levels showed a positive correlation with disease status. Additionally, we found a similar relationship between serum albumin levels and disease status (S1B and S2B Figs). On the other hand, there was no relationship between lipid parameters after treatment and disease status.

*14. Please provide Figure 5B*

→We appreciated this comment and corrected as the attachment.
